# Veratrum Viride in the Treatment of Acute Pneumonia

**Published:** 1887-05

**Authors:** James B. Baird

**Affiliations:** Atlanta, Ga.


### VERATRUM VIRIDE IN THE TREATMENT OF ACUTE PNEUMONIA.

BY JAMES B. BAIRD, M. D., ATLANTA, GA.

The inferior position assigned to veratrum viride as a therapeutic resource in acute pneumonia by many prominent writers and teachers must be surprising to those practitioners who have been in the habit of using the drug, and who have had favorable opportunities for observing its effects and of estimating correctly its real worth.

It is not infrequently incidentally mentioned in connection with the treatment of this affection, as a dangerous remedy, of uncertain power, that may be tried with great caution as a febrifuge in some cases not marked by depression, and which do not tend toward asthenia. While, in truth, discreetly used, it is neither dangerous nor depressing, and is most trustworthy and effective when applied to suitable cases. In my opinion, its therapeutic capabilities are not duly appreciated, and its dangers have been exaggerated.

By its judicious employment the action of the heart may be fixed at will ; and, while the number of pulsations may be reduced to any desired point, the strength of the systole, within reasonable limits, is apparently increased, rather than diminished.

It is especially in the earlier stages of the disease that the influence of the drug is so potent and so grateful, for, like the lancet, by reducing the heart's action, it withholds from the already surcharged lung the excessive flow of blood that a rapidly beating heart propels directly upon it ; but, unlike the lancet, it is not prodigal of the precious fluid, which is withheld, not wasted, and is saved to the system for future service, not sacrificed for a present need. In this view, then, veratrum may be regarded as a sustaining agent—the reverse of a depressant—as conserving the vital forces, not debilitating in its effect. It contributes to this result, not alone by obviating the demand for loss of blood by venesection, but also by relieving the heart from excessive and irritable action, thereby preventing exhaustion of the patient, and an unnecessary waste of the tissues.

It is by no means certain, moreover, that an impending or commencing attack of pneumonia may not be avertted or aborted by the prompt and vigorous use of veratrum and opium. I am aware that it is difficult, if not impossible, to establish the truth of a proposition of this character, but the weight of evidence, so far as my experience enables me to judge, favors the affirmative side of this question.

My practice has been to administer the tincture in doses of three, four or five drops to adults—and in exceptional cases even much larger doses may be confidently prescribed—combined with opium, at intervals of three or four hours, as required to accomplish and maintain the desired reduction in the pulse. Stimulants may, at the same time, be indicated. In my judgment they are generally needful at some period of an attack, and sometimes—always in the negro—they are absolutely indispensable. Brandy or whisky may, with perfect propriety, be administered in connection with the veratrum. No incompatibility exists between these remedies.

Their simultaneous exhibition is not inconsistent with sound therapeutic principles, and the practical results achieved sustain the wisdom of the procedure. Each fulfills its mission without conflict and without hindrance to the other.

An excessive dose of veratrum may cause nausea and vomiting, and urged recklessly it may, of course, produce alarming depression. The former effect, which the opium, under usual conditions, suffices to restrain, may be avoided by reducing the doses or by lengthening the intervals between them. The latter may be combatted, and with certainty corrected, by liberal stimulation.

To secure satisfactory results, the qua ity of the preparation dispensed should be above suspicion ; and if a pure and active specimen be secured a genuine pleasure is reserved for the physician who witnesses the gratifying sense of relief which its administration often brings to the laboring and sorely distressed patient.

The value of veratrum in many other diseases—notably those involving pulmonary and cerebral congestions — affords an inviting topic, and one worthy of serious consideration, but which could not be appropriately discussed in this brief contribution to an important subject.

